# A Novel Selective Sinus Reinforcement Technique for Acute Type A Aortic Dissection Involving the Aortic Root

**DOI:** 10.31083/RCM49343

**Published:** 2026-08-17

**Authors:** Hua-Jie Zheng, Ling-Ling Zeng, Yong-Bo Cheng, Mao-Ting Ye, Mei Guo, Ling-Feng Tang, Wei Cheng

**Affiliations:** ^1^Department of Cardiac Surgery, Southwest Hospital, Third Military Medical University (Army Medical University), 400038 Chongqing, China; ^2^Outpatient Department, Children’s Hospital of Chongqing Medical University, 400014 Chongqing, China

**Keywords:** aorta, thoracic, aortic valve, sinus of valsalva, aortic dissection

## Abstract

**Background:**

Acute type A aortic dissection (ATAAD) commonly involves the aortic root. We developed a novel selective sinus reinforcement (SSR) technique. This study aimed to investigate the feasibility, effectiveness and safety of this technique.

**Methods:**

Between January 2018 and January 2022, a total of 361 consecutive patients underwent surgery for ATAAD. Of these, 186 patients (51.5%) were treated using the SSR technique to repair the involved root.

**Results:**

The extent of sinus repair was stratified as follows: single sinus in 62.9%, two sinuses in 30.1%, and all three sinuses in 7.0%. Postoperatively, 5 patients (2.7%) required re-exploration for bleeding unrelated to the root procedure. At a mean follow-up of 35.2 ± 8.5 months, the 3-year overall survival rate was 94.6%. One patient required valve reintervention at 2 years due to late prolapse with severe aortic insufficiency.

**Conclusions:**

For ATAAD involving the root, the SSR technique offers a simplified and potentially safer surgical alternative, demonstrating durable aortic valve competence and excellent root stability.

## 1. Introduction

Acute type A aortic dissection (ATAAD) is a catastrophic cardiovascular emergency associated with high mortality and formidable surgical complexity [1]. In the surgical management of ATAAD with root involvement, the central challenge lies in determining the optimal extent of aortic replacement and selecting the most appropriate proximal root procedure [2]. The Bentall procedure remains an established benchmark for the surgical treatment of aortic root dissection [3]. Traditionally, the use of mechanical valves for aortic root replacement in patients under 55 years of age entails the requisite trade-off of lifelong anticoagulation [4]. In aortic dissection patients, anticoagulation can prevent false lumen thrombosis in the residual distal aorta, in addition to carrying bleeding and thromboembolic risks. Moreover, the durability of a bioprosthetic valve is a significant concern, especially in the young [5].

Given that the aortic annulus and leaflets are often unaffected in ATAAD, surgical efforts are increasingly directed toward preserving the native valve, particularly in young patients without leaflet pathology [6]. The surgical armamentarium for aortic root reconstruction has evolved from traditional techniques—which employed gelatin-resorcin-formalin glue, fibrin glue, or Teflon felt strips to reinforce the sinus—to sophisticated valve-sparing procedures such as the root remodeling (Yacoub) and reimplantation (David) operations [7,8,9,10]. Despite the established efficacy of these techniques, persistent challenges include the control of postoperative hemorrhage and the maintenance of the aortic root’s complex three-dimensional geometry. Moreover, in emergent settings, procedures such as the Yacoub and David operations can be technically complex and time-consuming, which may limit their applicability [11]. Thus, there remains a critical unmet need for innovative root repair solutions in emergency settings.

Some previous publications already indicated that the aortic wall dissection, when the root is involved, frequently does not extend throughout all 3 sinuses of Valsalva and, therefore, a replacement of the entire aortic root tissue is seldom necessary during valve-sparing surgery for ATAAD [12,13]. The non-coronary sinus (NCS) is the most commonly affected in the standard type of dissection, followed sequentially by the right and left coronary sinuses [14,15]. Consequently, in cases where pathology is confined to one or two sinuses, a targeted solution can avoid full root replacement. We therefore devised the selective sinus reinforcement (SSR) technique, which employs Dacron patches to selectively reconstruct the diseased sinuses. This preserves the integrity of uninvolved sinuses and obviates the need for coronary reimplantation. The present study was designed to evaluate the perioperative and follow-up outcomes of this valve-sparing SSR technique in patients with ATAAD.

## 2. Patients and Methods

This study was conducted in accordance with the Declaration of Helsinki (as revised in 2013) and was approved by the Institutional Review Board of Southwest Hospital of Third Military Medical University (Approval Number: KY2021058). The ethics committee specifically granted a waiver for obtaining written informed consent for the following reasons: (I) This was a retrospective study analyzing existing clinical data; (II) All patient data were anonymized and de-identified prior to analysis to protect privacy; (III) The research presented no more than minimal risk to the participants.

A total of 361 consecutive patients underwent open aortic repair for ATAAD at our center between January 2018 and January 2022. Among 288 patients identified with root involvement (defined as complete dissection of ≥1 sinus), surgical management was as follows: 100 underwent a Bentall procedure, 2 underwent a David V procedure, and the remaining 186 patients (51.5% of all ATAAD cases) formed the study cohort and were treated with the SSR technique to reconstruct all dissected sinuses.

Diagnosis of ATAAD was based on medical history, aortic computed tomography angiography (CTA), or magnetic resonance imaging and transthoracic echocardiogram (TTE).

### 2.1 Selective Sinus Reinforcement Technique

Patients were selected for the SSR technique only if all of the following criteria were met: (i) aortic sinus diameter <45 mm; (ii) Neri type A or B coronary lesion; (iii) a competent aortic valve without organic disease; and (iv) absence of connective tissue disease or a bicuspid aortic valve.

All procedures were performed through a median sternotomy. Cardiopulmonary bypass was established with arterial cannulation via the right axillary or femoral artery and venous drainage through bicaval cannulation. After systemic cooling to 30 °C, the ascending aorta was clamped and transected 1 cm above the sinotubular junction (STJ) to permit inspection of the root and aortic valve.

The aortic root was mobilized circumferentially down to the annulus, with mobilization of the coronary artery origins. After debridement of the false lumen, the aortic valve was resuspended by placing three 4-0 polypropylene pledgeted sutures at the commissural tops. Valve competence was assessed by saline injection, and concomitant cusp procedures (e.g., free margin reinforcement or patch plasty) were performed as needed. A Dacron graft (Vascutek Ltd., Inchinnan, Renfrewshire, UK) was sized to the diameter of the reconstituted sinotubular junction, achieved by upward traction on the commissural posts until the cusps coapted centrally. The graft diameter was selected to match this measured height, ensuring the creation of neo-sinuses of appropriate dimensions.

Reconstruction of the aortic sinus was determined according to the involvement of the aortic sinuses.

#### 2.1.1 Steps of the SSR Technique When the Dissection Involved Any Coronary Sinus and Non-coronary Sinus (NCS)

**Fig. 1.**
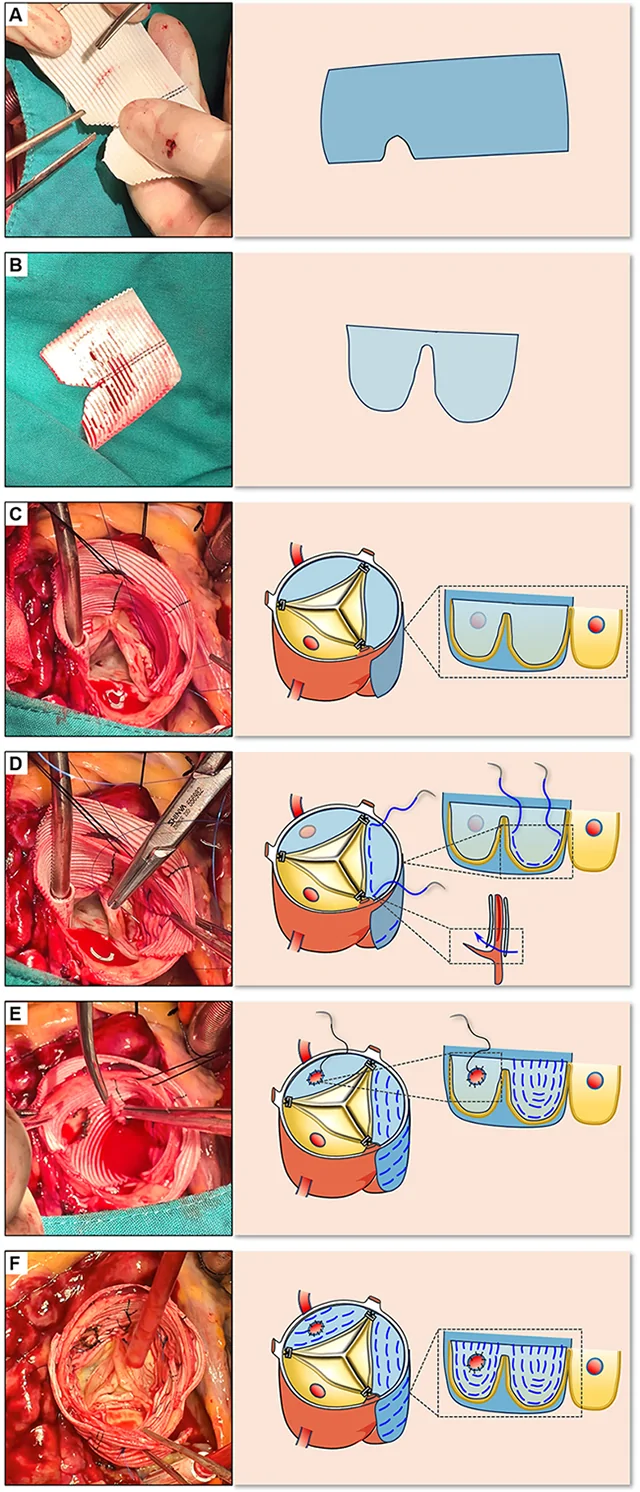
**Steps of the selective sinus reinforcement (SSR) technique for dissection involving a coronary sinus and the non-coronary sinus (NCS)**. (A) A rectangular Dacron patch is prepared and placed externally around the aortic root. A triangular opening was cut longitudinally at the level corresponding to the coronary arteries. (B) A single patch consisting of two interconnected U‑shaped sections is fashioned to match the dimensions of the involved coronary sinus and NCS. (C) The U‑shaped patch is inserted inside the affected sinuses as an internal liner, while the rectangular Dacron patch is positioned externally, creating a “sandwich” reinforcement around the native sinus wall. (D) The patches are secured to the aortic annulus using a running 5‑0 polypropylene suture, beginning at the sinus nadir and continuing toward the commissures. (E) A 3–4 mm circular opening is created in the coronary sinus portion of the internal patch using fine‑tip electrocautery. The coronary ostium and adjacent sinus wall are anastomosed to the margins of both internal and external patch openings with a running 6‑0 polypropylene suture. (F) The completed reconstruction shows the restored root with inner and outer Dacron layers, leaving unaffected sinuses intact.

(1) A Dacron patch for external root reinforcement was fashioned from the previously selected tubular graft. A 5 cm segment was excised from the graft, opened longitudinally, and flattened into a rectangular shape. The distance from the level of the aortoventricular junction to the inferior border of the coronary ostium was measured. A triangular opening was then cut longitudinally into the patch, extending from its base to the marked level of the coronary artery (Fig. 1A).

(2) A patch was excised from the uncrimped portion of the Dacron graft and fashioned into a bilobed, U-shaped structure to match the anatomical contours of the involved coronary and non-coronary sinuses (Fig. 1B). To ensure adequate coverage, the initial patch was deliberately cut generously; final trimming was performed during the suturing process to achieve an optimal fit.

(3) The native sinus wall was preserved. Reinforcement was achieved using a “sandwich” technique: the bilobed U-shaped patch was positioned inside the dissected coronary and non-coronary sinuses as an internal liner, while the rectangular Dacron patch was secured externally around the corresponding aortic segment (Fig. 1C). Meticulous placement was essential to avoid compromising the origins of the right and left coronary arteries.

(4) A running 5-0 polypropylene suture was used to secure the patches to the aortic annulus, starting at the nadir of each sinus and advancing toward the commissures (Fig. 1D). Sutures were placed along the stable cusp attachment line with stitches <3 mm apart to prevent puckering. Prior to final fixation, cusp coaptation was assessed with the commissural sutures untied. The continuous over-and-over suture technique reinforced the dissected wall, yielding a robust reconstruction.

(5) For an involved coronary sinus, a 3–4 mm opening was fashioned in the internal patch and carefully aligned with the native ostium and the external patch opening (Fig. 1E). The coronary orifice was then circumferentially sutured to the combined patch margins with a running 6-0 polypropylene suture.

(6) The reconstructed root now consisted of a robust, three-layer structure: the native aortic wall sandwiched between the inner and outer Dacron patches (Fig. 1F). The pre-selected Dacron tube graft was then anastomosed to the reconstructed root using a running 4-0 polypropylene suture. It is noteworthy that no fibrin sealants or biological glues were used in any patient in this series.

(7) Following core cooling to a rectal temperature of 25–27 °C, circulatory arrest was initiated. With clamp placement on the supra-aortic vessels, selective cerebral perfusion was delivered via the right axillary artery (10–15 mL/kg/min), enabling reconstruction of the aortic arch and distal aorta.

#### 2.1.2 Steps of the SSR Technique When the Dissection Involved Three Sinuses

**Fig. 2.**
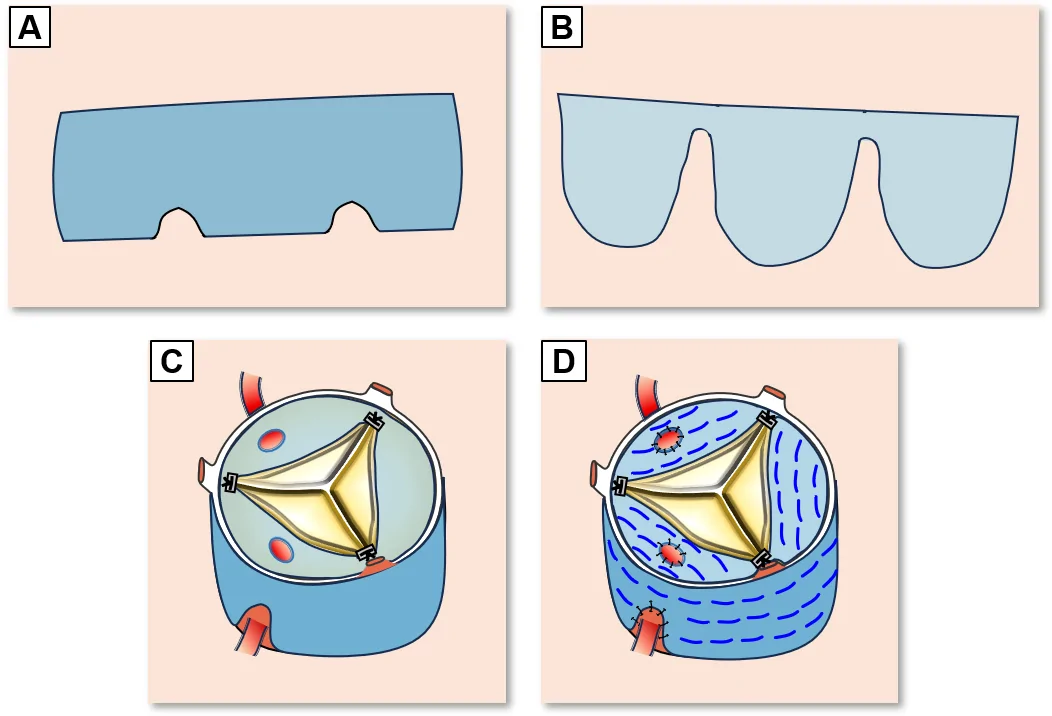
**Steps of the selective sinus reinforcement (SSR) technique for dissection involving all three sinuses**. (A) A rectangular Dacron patch is prepared and positioned externally around the aortic root. Triangular openings are created along the patch corresponding to the left and right coronary artery positions. (B) A single patch composed of three interconnected U‑shaped sections is fashioned to match the size and contour of each sinus. (C) The tripartite U‑shaped patch is deployed inside the sinuses as an internal liner, while the rectangular patch is wrapped externally around the root, forming a three‑layer reconstruction. (D) Coronary ostial reconstruction is performed using the technique previously described, with circular openings created in the internal patch and anastomosed to the coronary ostia using a running 6‑0 polypropylene suture.

(1) A rectangular Dacron patch was prepared from a 5-cm graft segment. Based on the measured distances from the aortoventricular junction to each coronary ostium, two triangular openings were meticulously created from the patch’s base, corresponding to the positions of the left and right coronary arteries (Fig. 2A).

(2) A patch was excised from the uncrimped portion of the Dacron graft and fashioned into a single unit comprising three interconnected U-shaped sections, designed to match the anatomical contours of all three sinuses of Valsalva (Fig. 2B).

(3) The triple U-shaped patch was deployed within the respective sinuses as an internal liner, while the rectangular Dacron patch was secured around the external surface of the root, completing the three-layered reconstruction (Fig. 2C).

(4) The methods for reinforcing the aortic sinus and operating on the coronary artery openings are exactly the same as those mentioned above (Fig. 2D).

#### 2.1.3 Steps of the SSR Technique When an Intimal Tear Only Existed in the Non-coronary Sinus

**Fig. 3.**
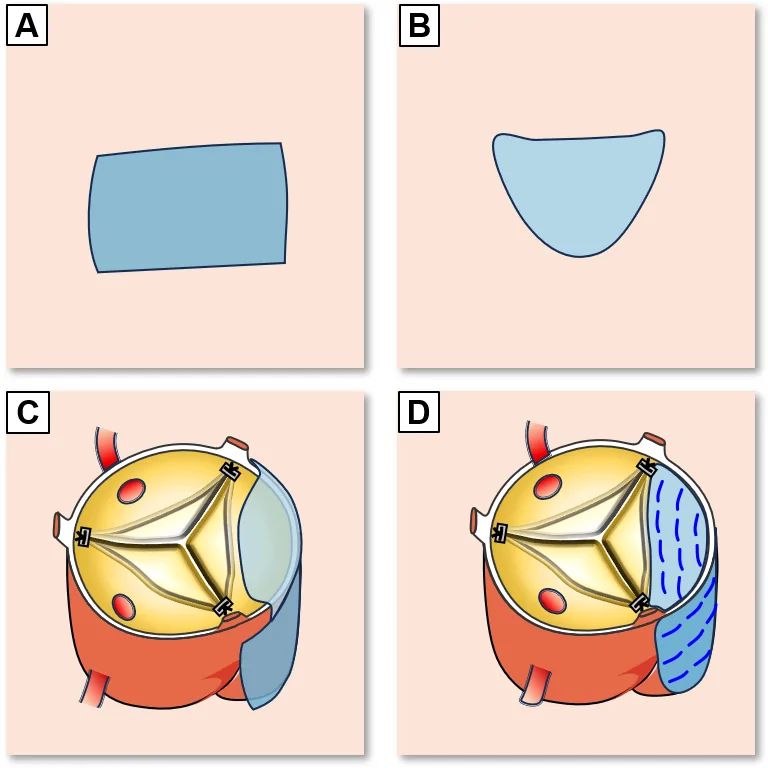
**Steps of the selective sinus reinforcement (SSR) technique for dissection localized to the non-coronary sinus (NCS)**. (A) A segment of cylindrical Dacron graft (~5 cm in length) is opened longitudinally to form a rectangular patch for external reinforcement. (B) A separate U‑shaped patch is trimmed to match the anatomical dimensions of the non-coronary sinus. (C) The U‑shaped patch is positioned inside the NCS as an internal support, while the rectangular patch is secured externally over the same sinus. (D) The sinus is reconstructed using the previously described suturing technique, with no need for coronary reimplantation in this setting.

(1) A 5-cm segment of a cylindrical Dacron graft was incised longitudinally and fashioned into a rectangular patch (Fig. 3A).

(2) One patch was excised from the uncrimped part of the Dacron tube and trimmed into a U-shaped structure, compatible with the size and shape of the NCS (Fig. 3B).

(3) The U-shaped patch was inserted within the NCS, and the rectangular Dacron patch was correspondingly placed around the outer surface of the NCS (Fig. 3C).

(4) The methods for reinforcing the NCS was exactly the same as those mentioned above, without a coronary manipulation (Fig. 3D). This approach is technically less complex than reconstruction involving a coronary sinus.

### 2.2 Transesophageal Echocardiography

Transesophageal echocardiography (TEE) was performed in all patients to assess aortic valve regurgitation, leaflet morphology (e.g., prolapse, calcification, or thickening), and the proximal extent of the dissection. A comprehensive intraoperative protocol included TEE at three time points: after anesthesia induction (before skin incision), following root repair (before weaning from cardiopulmonary bypass), and after hemodynamic stabilization (before sternal closure). Aortic insufficiency (AI) was quantified by color flow mapping and continuous-wave Doppler, and graded as none (grade 0), trace (0.5+), mild (1+), moderate (2+), moderate-severe (3+), or severe (4+).

### 2.3 Follow-Up

Baseline, perioperative, and follow-up data were retrospectively collected from our institutional database. The primary endpoints were defined as all-cause mortality, valve reintervention, aortic reintervention, and the grade of AI.

All patients were followed according to a standardized protocol. Echocardiography was recommended at discharge, 6 months, 12 months, and annually thereafter. In patients with residual distal dissection, CTA was additionally performed at the same intervals. Examinations were conducted either at our outpatient clinic or by the patient’s local cardiologist. For this study, follow-up data were systematically collected by telephone contact with patients and their cardiologists, and all available echocardiographic and CTA records were retrieved for centralized review. Follow-up continued until the occurrence of death from any cause or loss to follow-up. Definitions of neurological outcomes: Permanent neurological deficit was defined as a focal deficit persisting >72 hours with imaging confirmation. Temporary neurological dysfunction was defined as confusion, delirium, or agitation lasting >48 hours without focal signs or imaging correlate.

### 2.4 Statistical Analysis

Continuous variables were presented as mean (± standard deviation) when conforming to normal distribution, or as median with interquartile range (25th–75th percentiles); categorical variables were presented using absolute and relative frequencies. Overall survival was estimated by the Kaplan-Meier method starting with the procedure. The statistical analyses were performed using SPSS software (version 26; IBM, Armonk, NY, USA). All analyses were two-tailed, and *p* values < 0.05 were considered statistically significant.

## 3. Results

### 3.1 Baseline Characteristics

Preoperative clinical characteristics of patients are shown in Table 1. The study cohort had a mean age of 46.5 ± 11.0 years (range, 27–63) and was predominantly male (131 patients, 70.4%). Preoperatively, moderate or severe aortic insufficiency (≥grade 2+) was present in 105 patients (56.5%). The mean size of preoperative aortic annulus, sinuses of Valsalva (SOV) and left ventricular end diastolic diameters (LVEDD) was 24.7 ± 2.0 mm, 37.0 ± 2.1 mm, and 50.0 ± 6.6 mm, respectively.

**Table 1.** **Preoperative characteristics of patients undergoing selective sinus reinforcement (SSR)**.

Variable		All (n = 186)
Age (years)		46.5 ± 11.0
Female		55 (29.6)
Smoking		98 (52.7)
Hypertension		159 (85.5)
Diabetes		9 (4.8)
Chronic kidney disease		10 (5.4)
eGFR (mL/min/1.73 m^2^)		39.2 ± 20.4
Hemoglobin (g/dL)		12.5 ± 1.9
History of stroke		6 (3.2)
Peripheral arterial disease		9 (4.8)
COPD		20 (10.8)
Coronary artery disease		6 (3.2)
Malperfusion syndrome		
	Brain	5 (2.7)
	Coronary artery	5 (2.7)
	Renal	9 (4.8)
	Superior mesenteric artery	2 (1.1)
	Lower extremity	9 (4.8)
Aortic insufficiency		
	0 (None)	6 (3.2)
	0.5 (Trace)	25 (13.4)
	1 (Mild)	50 (26.9)
	2 (Moderate)	50 (26.9)
	3 (Moderate-severe)	22 (11.8)
	4 (Severe)	33 (17.7)
Aortic annulus (mm)		24.7 ± 2.0
SOV (mm)		37.0 ± 2.1
STJ (mm)		34.3 ± 3.1
Ascending aorta (mm)		43.5 ± 5.2
LVEDD (mm)		50.0 ± 6.6
LVEF (%)		54.5 ± 8.9

Data are presented as number (% of group total), or mean ± standard deviation. COPD, chronic obstructive pulmonary disease; eGFR, estimated glomerular filtration rate; LVEDD, left ventricular end-diastolic diameter; LVEF, left ventricular ejection fraction; STJ, sinotubular junction diameter; SOV, sinuses of Valsalva.

### 3.2 Operative Details

Operative details are provided in Table 2. Cardiopulmonary bypass and aortic cross-clamp time were 156 ± 32 minutes and 93 ± 16 minutes, respectively. The distribution of sinus reinforcement was: a single sinus in 62.9% (n = 117; n = 111 non-coronary, n = 6 right coronary) and two sinuses in 30.1% (n = 56; n = 51 both right and non-coronary, n = 5 both left and non-coronary), and all three sinuses in 7.0% (n = 13).

**Table 2.** **Operative variables**.

Variable			All (n = 186)
Cardiopulmonary bypass time (min)			156 ± 32
Aortic cross-clamp time (min)			93 ± 16
Total procedure time (min)			271 ± 38
One sinus reinforcement			117 (62.9)
	NCS		111 (59.7)
	RCS		6 (3.2)
Two-sinus reinforcement			56 (30.1)
	RCS+NCS		51 (27.4)
	LCS+NCS		5 (2.7)
Three-sinus reinforcement			13 (7.0)
Concomitant procedures			
	Partial aortic arch replacement		41 (22.0)
	Complete aortic arch replacement		145 (78.0)
	CABG		6 (3.2)
	Valve repair		125 (67.2)
		Commissuroplasty	58 (31.2)
		Cusp plication/shortening	26 (14.0)
		Combination	41 (22.0)
Chest closure AI			
	0 (None)		86 (46.2)
	0.5 (Trace)		95 (51.1)
	1 (Mild)		5 (2.7)
	2 (Moderate)		0
	3 (Moderate-severe)		0
	4 (Severe)		0

Data are presented as number (% of group total), or mean ± standard deviation. AI, aortic insufficiency; CABG, coronary artery bypass grafting; LCS, left coronary sinus; NCS, non-coronary sinus; RCS, right coronary sinus.

Concomitant valve repair was performed in 125 patients (67.2%), involving commissuroplasty (n = 58), cusp free margin plication/shortening (n = 26), or a combination of techniques (n = 41). Our intraoperative protocol mandated repeat cross-clamping for any patient with more than mild AI (>1+) upon weaning from cardiopulmonary bypass. This aggressive approach resulted in excellent valve function at the end of the procedure, with nearly all patients (n = 181, 97.3%) demonstrating none or trace AI (0–0.5+) on transesophageal echocardiography at chest closure. Only five patients (2.7%) had mild (1+) AI at that time.

### 3.3 Postoperative Outcomes

Postoperative outcomes are presented in Table 3. No anastomotic bleeding occurred. Re-exploration for bleeding was required in 5 patients (2.7%), none of which was related to the aortic root. Other complications included temporary neurological dysfunction (confusion, delirium, or agitation lasting >48 hours without focal signs) in 5 patients (2.7%), and respiratory failure requiring prolonged mechanical ventilation (>48 hours) in 22 patients (11.8%), which was the most frequent complication. Acute renal failure occurred in 9 patients (4.8%), all of whom recovered completely within 2–3 months. Notably, no patient experienced permanent stroke (permanent neurological deficit defined as focal deficit persisting >72 hours with imaging confirmation) or required permanent pacemaker implantation.

**Table 3.** **Postoperative outcomes**.

Variable		All (n = 186)
Pericardial drainage volume in 24 h after surgery (mL)		550 ± 103
Re-exploration for bleeding		5 (2.7)
Postoperative neurological complications		5 (2.7)
	Permanent neurological deficit	0
	Temporary neurological dysfunction	5 (2.7)
PPM placement		0
Acute renal failure		9 (4.8)
Prolonged ventilation (>48 hours)		22 (11.8)
Intensive care unit length of stay (hours)		62.5 ± 29.6
Hospital length of stay (days)		11.7 ± 2.5
In-hospital mortality		6 (3.2)

Data are presented as number (% of group total), or mean ± standard deviation. PPM, permanent pacemaker.

#### 3.3.1 Survival

**Fig. 4.**
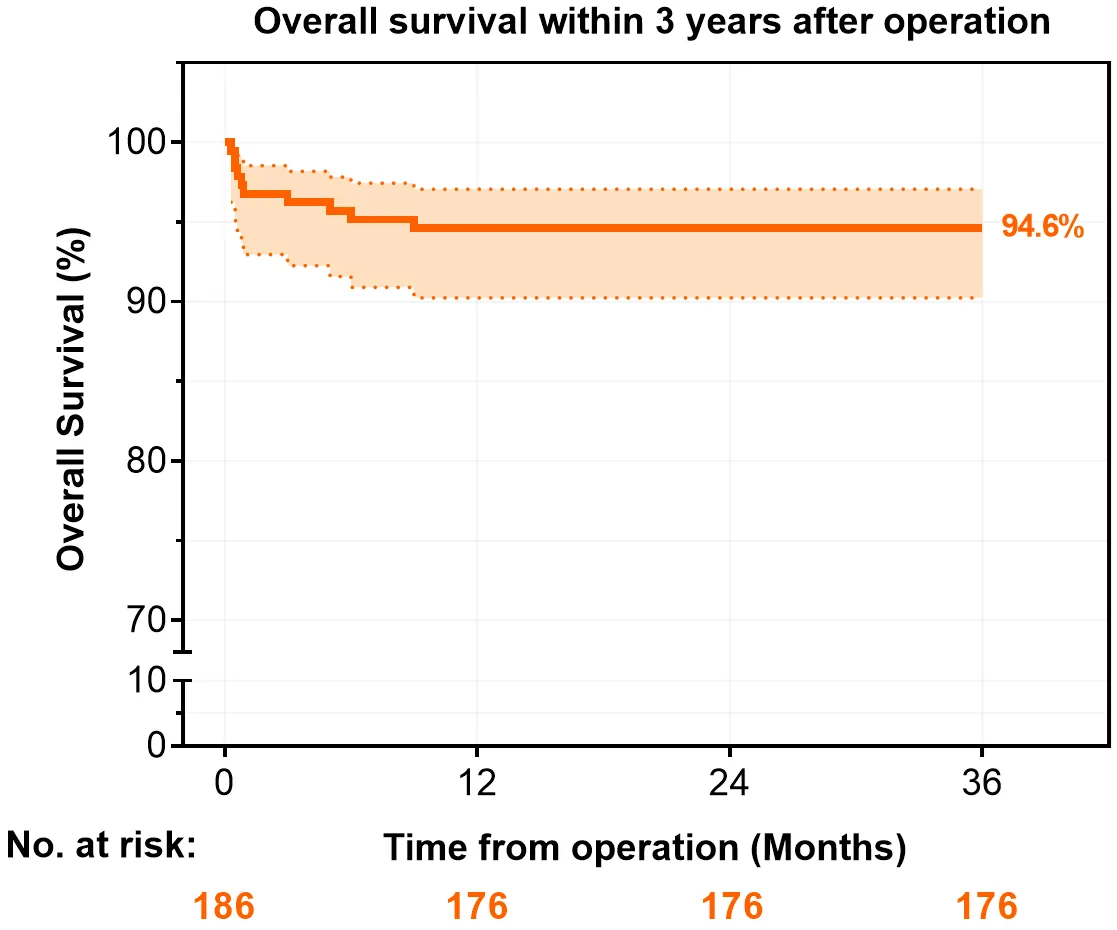
**Overall survival after the selective sinus reinforcement for valve-sparing aortic root repair in acute type A dissection**. Dotted lines indicate 95% confidence intervals, with numbers at the bottom of the figure showing numbers at risk for the given time points.

The mean follow-up duration was 35.2 ± 8.5 months, and no patients were lost to follow-up. A total of 10 patients died during hospitalization (n = 6, 3.2%) or follow-up (n = 4, 2.2%). Overall survival at 3 years after surgery was 94.6% (Fig. 4). Causes of in-hospital mortality included gastrointestinal tract necrosis (n = 1), gastrointestinal hemorrhage (n = 1), sepsis (n = 1), multisystem organ failure (n = 2), and descending aortic rupture (n = 1). During follow-up, deaths resulted from a ruptured abdominal aortic aneurysm (n = 1), acute myocardial infarction (n = 1), pancreatic cancer (n = 1), and a traffic accident (n = 1). Critically, no mortality was attributed to dysfunction of the reconstructed aortic root or valve.

#### 3.3.2 Valve-Related Morbidity and Reoperation

**Fig. 5.**
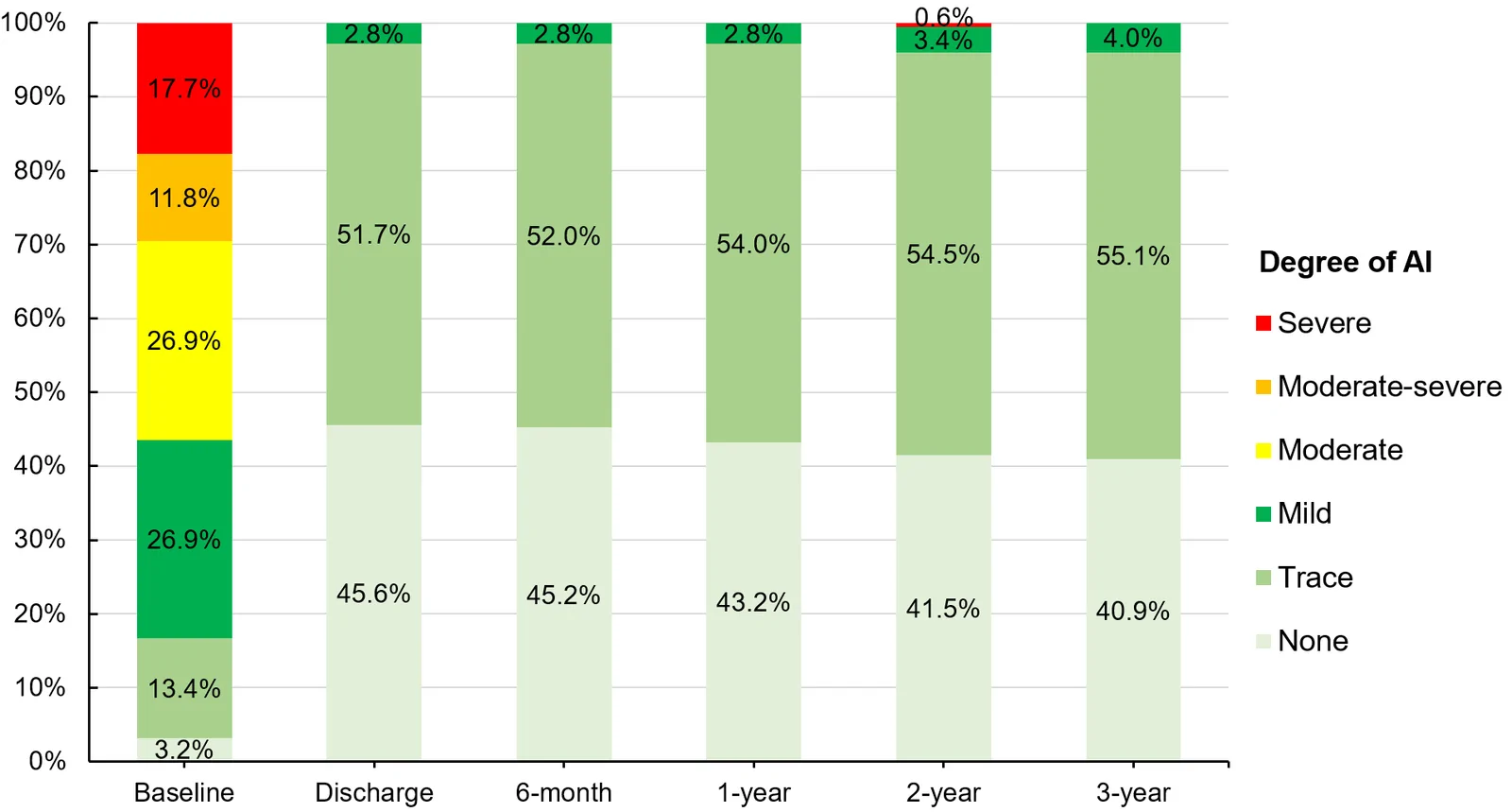
**Echocardiographic evidence of aortic insufficiency (AI) in each patient at the time points**.

Follow-up echocardiography was performed in all survivors. No evidence of aortic stenosis was detected. Degree of AI at each time point is shown in Fig. 5. Only one patient required late aortic valve reintervention. This patient, discharged on postoperative day 10 with an uncomplicated initial recovery, developed severe AI due to late-onset leaflet prolapse at 2 years. Following reoperation, she achieved an excellent clinical outcome with only trace residual AI. There was no documented valve thrombosis at follow-up.

No patient required reoperation on the proximal aorta across follow-up. All aortic reinterventions were distal (n = 10; 8 endovascular and 2 open thoracoabdominal aortic replacement). There were no deaths due to these additional reoperations.

**Fig. 6.**
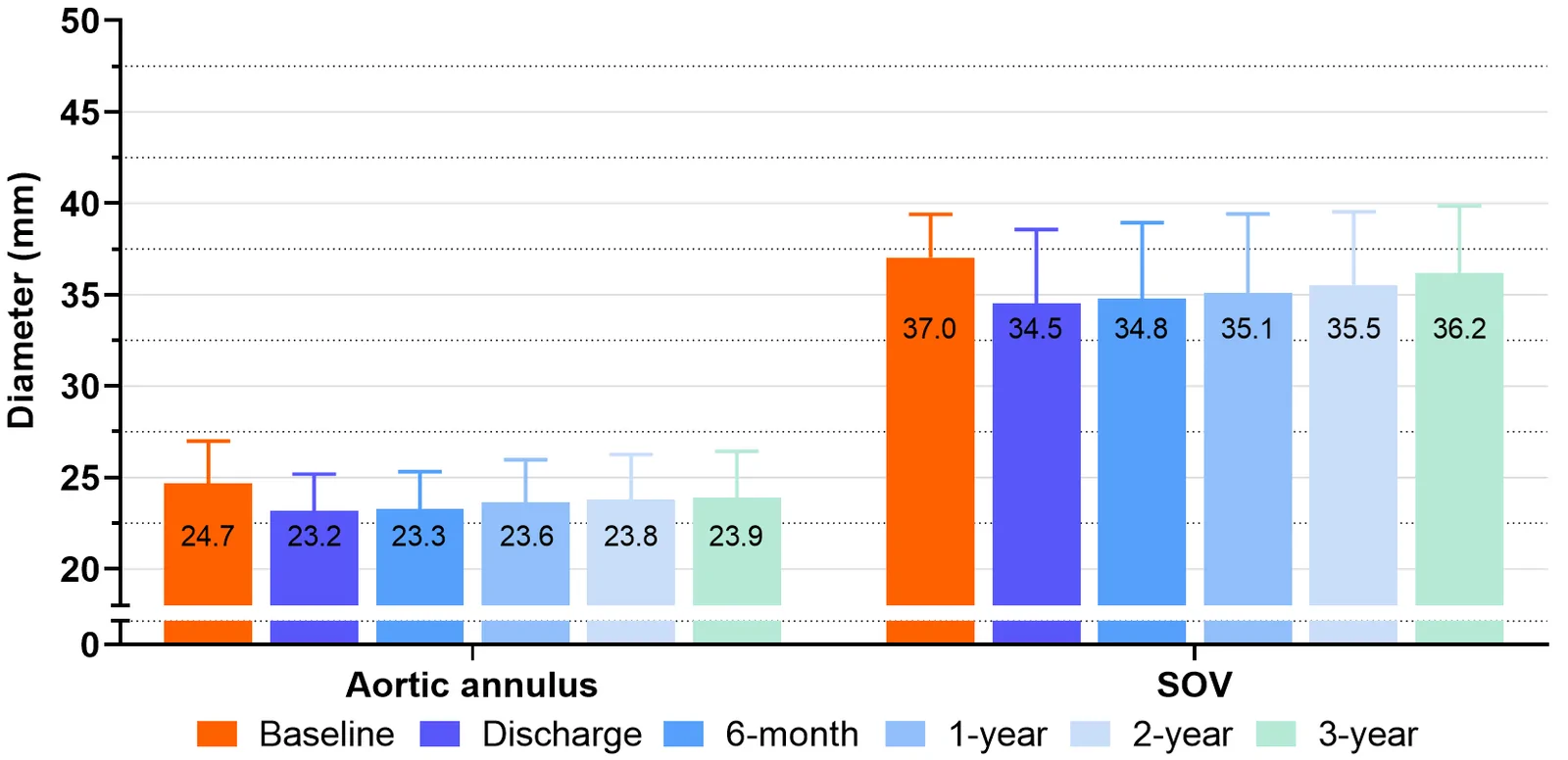
**The diameters of aortic root at each follow-up time point**. SOV, sinuses of Valsalva.

The diameters of aortic root at each follow-up time point are shown in Fig. 6. Aortic root dimensions remained stable in all cases during the entire follow-up time (annulus growth rate: +0.29 ± 0.17 mm/year; SOV: +0.36 ± 0.21 mm/year).

## 4. Discussion

The primary aim of surgery in the treatment of ATAAD is to prevent rupture of the dissection and subsequent hemorrhage. For patients with involvement of the aortic root, there are two conventional methods of surgical management. First, if the aortic root has evidence of aortic valve or aortic ring pathologies, or there is an existing aortic aneurysm, a valve sparing [16] or Bentall approach [17] may be used. Second, if the aforementioned pathologies are not apparent, ascending aortic replacement with traditional aortic root reconstruction (supracommissural replacement) may be performed [18]. Various modifications of aortic valve sparing approaches have also been described, including Teflon remodeling [19], gluing dissected layers [20], and supracoronary replacement of the ascending aorta with root reconstruction [21]. However, there is a lack of consensus as to the optimal surgical approach for the treatment of ATAAD involving the aortic root, which provokes a further search for new surgical solutions.

We developed a novel selective sinus reinforcement (SSR) technique in which Dacron patches are used to reconstruct both the inner and outer layers of the dissected sinus, creating a “sandwich” reinforcement around the native sinus wall. Rather than resecting the compromised tissue, the dissected sinus is stabilized by securing tailored U‑shaped patches along its internal and external surfaces. Although the external root diameter is often enlarged in ATAAD, the underlying architecture of the aortic root typically remains structurally intact. By anchoring the patches along the native anatomic contours, the normal three‑dimensional geometry of the root is reliably restored—a critical factor in ensuring the long‑term function and durability of the preserved native valve.

The SSR technique enables selective replacement of only the diseased sinuses while preserving the normal or near-normal sinuses in patients with ATAAD and a non-dilated aortic root. In our cohort, fewer than 10% of patients required replacement of all three sinuses. By limiting repair to the pathologically involved sinuses of Valsalva, the SSR technique is associated with operative and cross-clamp times as reported and obviates unnecessary coronary reimplantation from uninvolved sinuses—thereby mitigating associated risks. Notably, no coronary ostial complications related to patch implantation occurred in this series. This approach facilitates an individualized, case-based reconstruction strategy that restores functional anatomy while leaving healthy sinus architecture intact.

In both Yacoub’s remodeling technique [22] and David’s reimplantation procedure [23], the extensive suture line between the native aortic remnant and the Dacron graft lies deep in the surgical field, making hemostatic control challenging in the event of anastomotic bleeding. In contrast, the SSR technique positions reinforcing patches directly against the involved sinus and secures them along the aortic valve annulus. This design provides a clearer operative field and enables more precise suture placement, thereby facilitating hemostasis and simplifying the operative procedure. Consistent with this technical advantage, none of the 5 patients (2.7%) in this series who required re-exploration for bleeding had hemorrhage originating from the reconstructed root.

Our overall survival rate compares favorably with those reported in previous studies after aortic root reconstruction with valve sparing, in which the 5-year survival rates were found to be 89.4 ± 3.4% [24]. Our in-hospital mortality rate (3.2%) also compares favorably to that associated with supracommissural replacement, which typically ranges from 6% to 10% [25,26]. However, direct comparison with historical data is subject to bias, and we have not claimed superiority.

Valve durability remains a primary concern in valve-sparing aortic root procedures. In this series with 3-year follow-up, significant aortic regurgitation was uncommon. Only one patient developed severe AI due to late leaflet prolapse at 2 years, which was successfully addressed by reoperation. The patient currently maintains trace AI and excellent clinical status, supporting the potential for durable functional outcomes with the SSR technique.

Rate of freedom from aortic root reoperation in patients who have undergone prior aortic repair for ATAAD with root retention is a very important factor and has been reported to be between 81 % and 96% at 5 years [16,27,28,29]. It is well known that most of dilatation of the aortic annulus occurs beneath the commissures of the non-coronary cusp [30]. The internal and external reinforcement in SSR technique ensures sustained stability of the non-coronary sinus annulus, which may protect against late dilatation. This is corroborated by our 3-year follow-up, which showed no reoperations for root recurrence and only negligible root growth, supporting its use as a safe and effective option for root involvement in ATAAD.

## 5. Limitations

Study limitations. This study has several notable limitations. First, the lack of an internal control group limits the ability to draw comparative conclusions. The SSR and Bentall groups had non‑overlapping indications (sinus diameter ≥45 mm, connective tissue disease, bicuspid valve, or organic valve disease for Bentall vs. absence of these for SSR), making direct comparison subject to severe selection bias. Second, selection bias is inherent to our inclusion criteria (sinus <45 mm, competent valve, no connective tissue disease, no bicuspid valve), which select patients with less severe root pathology; thus, favorable outcomes should be interpreted only in this context and cannot be generalized to all ATAAD patients with root involvement. Third, the relatively short follow-up (mean 35.2 months) limits conclusions about long-term durability. Fourth, low event rates (10 deaths, 1 valve reintervention) precluded multivariable analysis. Fifth, potential late complications include progressive root dilatation or aneurysm formation, risk of late redissection or structural failure at the Dacron‑native aortic interface, possible biomechanical alterations due to patch reinforcement that may affect root dynamics over time, and theoretical risk of pseudoaneurysm formation. Continued annual surveillance with echocardiography and CTA is essential. Future studies with extended follow-up, larger cohorts, and propensity-matched comparisons are warranted.

## 6. Conclusions

For ATAAD involving the root, the SSR technique offers a simplified and potentially safer surgical alternative, demonstrating durable aortic valve competence and excellent root stability.

## Data Availability

The data that support the findings of this study are available from the corresponding authors upon reasonable request.
